# Editorial for Special Issue “Microbial Safety and Beneficial Microorganisms in Foods”

**DOI:** 10.3390/microorganisms14051032

**Published:** 2026-05-01

**Authors:** Theodoros Varzakas

**Affiliations:** Department of Food Science and Technology, University of the Peloponnese, 24100 Antikalamos, Greece; t.varzakas@uop.gr

The role of microorganisms in food was first acknowledged a long time ago. Detrimental effects on food systems have been reported from pathogenic microorganisms, such as *Salmonella* and *Escherichia coli* causing severe foodborne illnesses. On the other hand, beneficial microorganisms have been shown to enhance food stability, safety, and nutritional value, and they are particularly found in fermented foods and biopreservation.

This Special Issue provides a platform for researchers to enrich their knowledge on microbial safety and the beneficial use of microorganisms in food. In this Special Issue, six research contributions have been included, along with four review papers.

In the first contribution, we addressed antimicrobial resistance (AMR) and the role of artisanal dairy fermentations (Tyrovolia–Kopanisti) in enriching resistance phenotypes and serving as sensitive sentinels of AMR dynamics.

In the second contribution, it was shown that the fermentative performance of amaranth seeds improved through sprouting and probiotic fermentation, incorporating potential health-promoting properties.

The safety profile of *Lentilactobacillus buchneri*, which has neuroprotective effects, needs to be evaluated through both phenotypic and genotypic analyses for suitability in probiotic applications, as discussed by Kim et al. (contribution 3).

A rapid, robust, and scalable tool for precise probiotic strain tracking and accurate quantification of *Bifidobacterium animalis* subsp. *lactis* has been addressed, enabling critical quality control and meeting regulatory compliance needs (contribution 4).

Waste bread (WB) as a novel, cost-effective, and nutrient-rich substrate for microbial growth has been highlighted in contribution 5. It acts as a fermentation substrate to enhance amylase production, offering sustainable development.

The last research contribution (contribution 6) deals with fucoidan as a natural antibacterial agent for controlling *Salmonella enterica* subsp. *Enterica* Serovar Typhimurium biofilms in seafood processing. Nowadays, enhancement of food safety and minimization of contamination are essential strategies to improve quality of life.

The first review (contribution 7) deals with probiotics selection and precise strain selection, novel encapsulation technologies for viability, and strict safety assessments as described by EFSA and the United States FDA. Unlocking next-generation probiotics depends on omics technologies, synthetic biology, and more detailed microbiome–host interaction studies.

Contribution 8 examines the One Health framework, in terms of the drivers of AMR across sectors, focusing on surveillance systems and regulatory policies aiming at ensuring the microbiological safety of food systems.

Elbehiry et al. (contribution 9) discussed cutting-edge control strategies, such as natural antifungal agents, essential oils, biocontrol methods, and non-thermal technologies like cold plasma and UV-C treatment, along with safe microbial control approaches in fresh produce.

Finally, contribution 10 provided an overview of post-harvest food decontamination methods against some of the most important bacterial foodborne pathogens, focusing on the advantages and challenges of phages and phage-added packaging materials.

